# Phylogenetic diversity of actinomycetes cultured from coastal multipond solar saltern in Tuticorin, India

**DOI:** 10.1186/2046-9063-8-23

**Published:** 2012-09-05

**Authors:** Polpass Arul Jose, Solomon Robinson David Jebakumar

**Affiliations:** 1 Department of Molecular Microbiology, School of Biotechnology, Madurai Kamaraj University, Madurai, 625 021, India

**Keywords:** Solar saltern, Actinomycete, ARDRA, Phylogeny, 16S rDNA, *Nonomuraea*

## Abstract

**Background:**

Hypersaline solar salterns are extreme environments in many tropical and subtropical regions throughout the world. In India, there are several coastal solar salterns along with the coastal line of the Bay of Bengal and Arabian Sea and inland solar salterns around Sambhar saltlake, from which sodium chloride is obtained for human consumption and industrial needs. Studies on characterization of such coastal and inland solar salterns are scarce and both the bacterial and archaeal diversity of these extreme saline environment remains poorly understood. Moreover, there are no reports on exclusive diversity of actinomycetes inhabiting Indian solar salterns.

**Results:**

Soil sediments were collected from both concentrator and crystallizer ponds of solar salterns and subjected to detailed physico-chemical analysis. Actinomycetes were selectively isolated by employing selective processing methods and agar media. A total of 12 representatives were selected from the 69 actinomycete isolates obtained from the saltern soil samples, using Amplified Ribosomal DNA Restriction Analysis. Sequencing and analysis of 16S rDNA from chosen representative isolates displayed the presence of members affiliated to actinobacterial genera: *Streptomyces*, *Micromonospora, Nocardia, Nocardiopsis, Saccharopolyspora* and *Nonomuraea.* The genus *Streptomyces* was found to be the dominant among the isolates. Furthermore, rare actinomycete genus *Nonomuraea* was isolated for the first time from Indian solar salterns.

**Conclusions:**

To the best of our knowledge, this study constitutes the first characterization of actinomycete diversity centred on solar salterns located in the eastern coastal region of India. Furthermore, this is the very first report of isolation of *Nonomuraea* species from solar salterns and also from India. As actinomycetes encompass recurrently foremost sources of biotechnologically important member of the microbial communities, the actinomycetes retrieved from the Indian saltern soil samples laid the platform to search for novel biotechnologically significant bioactive substances.

## Background

Actinomycetes are outstanding collection of microorganisms with numerous affiliates reported for different kinds of bioactive features and extensive commercial importance [1,2]. Several reports on the ecology of actinomycetes have described that these microorganisms are widespread in nature and may occur in extreme environments. Accordingly, groups of acidophilic and alkaliphilic, psychrophilic and thermophilic, halophilic and haloalkaliphilic, and xerophilic actinomycetes have been described [3,4]. The halophilic actinomycetes are noteworthy for their potential to produce bioactive compounds and enzymes [5,6]. In recent years*,* novel actinomycetes of diverse genera: *Nocardiopsis, Saccharopolyspora*, *Myceligenerans* and *Streptomyces* have been isolated from hypersaline environments [7-11]. Despite these findings, little is known about the diversity of actinomycetes in hypersaline environments. Search for novel and biotechnologically exploitable microorganisms has motivated researchers to screen largely unexplored extreme environments in which some chemical or physical factors differ considerably from that found in habitats which support human life. Moreover, the exploration of untapped actinomycetes diversity is a key practice for hunting novel taxa and genes of value to biotechnology [12,13].

The solar salterns are hypersaline environments that consist of a series of shallow ponds connected in a sequence of increasing saline brines that are used for the commercial production of salt from seawater [14]. Sequential precipitation of calcium carbonate, calcium sulfate and sodium chloride occurs during the evaporation of seawater. India is the third largest salt producing country in the world with an extent of about 5.50 lakh acres engaged in salt production. The coastal solar salterns accounts for about 91% of the country’s salt production and inland salterns accounts for remaining 9% of production. There have been several investigations on microbial assemblages in such hypersaline solar salterns [15,16]. The diversity of these microbial assemblages in hypersaline environments has been studied by employing both molecular and culture-dependent methods [17-20]. Previous studies of Indian solar salterns have been focused on the archeal [21] and Cyanobacterial [22] diversity. The aims of the present work were to use selective processing methods to isolate actinomycetes from coastal solar saltern ponds and to determine their phylogenetic diversity.

## Results

### Physico-chemical characteristics of saltern soil

The physico-chemical characteristics of soil samples collected from both concentrator and crystallizer ponds of solar salterns at Tuticorin, India were studied using standard protocols and summarized in Table 1. Several parameters, such as pH, electric conductivity, concentration of macroelements and trace elements were measured. The pH was just above neutral in both concentrator and crystallizer ponds and negligible variation was found between the ponds. Likewise, the EC values of concentrator and crystallizer pond soil samples were found to be 12.75 and 12.92, respectively. This moderate EC values conforms the hypersaline nature of both soil samples. Sodium and potassium were found to be dominant in both ponds.

**Table 1:** Physico-chemical parameters observed in the soil samples collected from both Concentrator and Crystallizer ponds of coastal solar saltern

**Parameters**	Values (ppm)*	
	**Concentrator Pond**	**Crystallizer Pond**
pH*	8.15	8.27
EC (dSm-1)*	12.75	12.92
T. Sodium	3340	3840
T. Nitrogen	140	130
T. Phosphorus	240	240
T. Potassium	460	460
Organic C	1500	1200
Fe	35.44	28.88
Mn	11.16	15.44
Zn	23.8	4.44
Cu	1.5	1.3

*Values are in ppm except for pH and EC.

### Selective isolation of actinomycetes

A total of 69 actinomycete isolates were obtained from saltpan soil samples using two sample processing methods and four different isolation media. Among the two sample-processing methods, stamping dried sediments onto low-nutrient agar (IM4) resulted in the isolation of 68% of actinomycete strains which proved to be a highly successful method to cultivate actinomycete and the result is in accordance with the earlier report of Gontang *et al*. [23].

### 16S rDNA amplification and ARDRA

**Figure 1:**
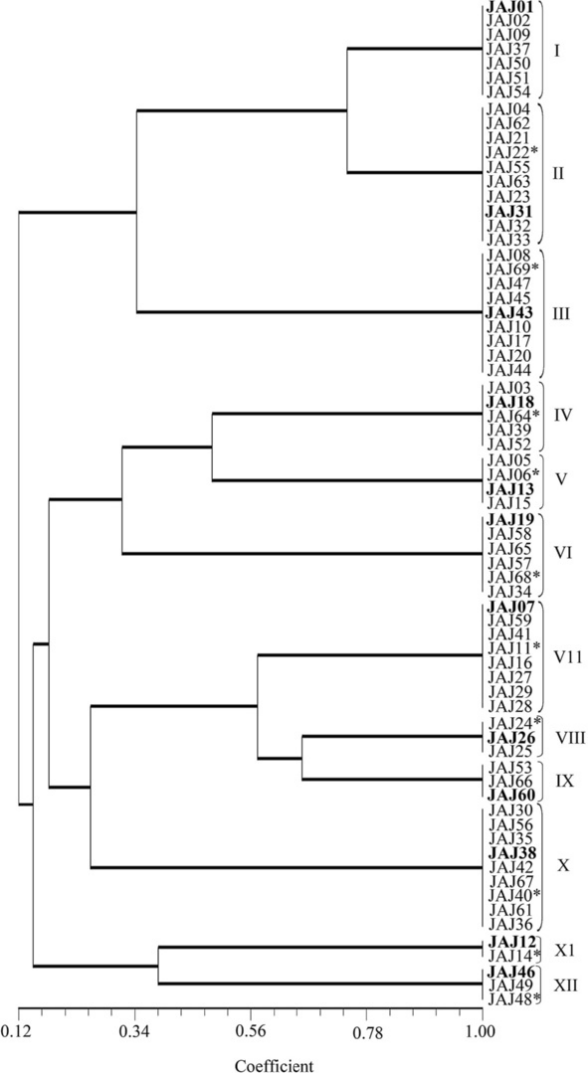
**UPGMA dendrogram inferred from ARDRA patterns of actinomycetes isolated from solar saltern ponds.** The dendrogram shows the clustering of 69 actinomycete isolates generated from amplified ribosomal DNA restriction analysis with restriction endonuclease *HaeIII,* using the UPGMA algorithm and the Jaccard’s coefficient. The Roman numerals I to XII represent the twelve clusters obtained in the analysis. The ribotypes selected from sequencing analysis are highlighted in boldface. The additional isolates subjected to sequence analysis are starred.

PCR amplification of 16S rDNA using a set of universal eubacterial specific primers: 27 F and 1492R yielded a single amplicon of ~1500 bp for all the isolates. Restriction digestion of these amplicons with *HaeIII* yielded different profiles characterized by three to four fragments ranging from 100 bp to 700 bp in size for the different isolates. When amplicons were digested with restriction enzyme *TaqI*, almost similar profile was obtained for all the isolates. The actinomycete isolates were divided into of 12 groups in a dendrogram (Figure 1) inferred from ARDRA patterns obtained with *HaeIII*. Group I has 7 isolates, Groups VII, IX and XII have 3 isolates each. The groups II, XI, VII, IV, VI and X have 10, 2, 8, 5, 6 and 9 isolates, respectively.

### Actinomycete community composition and phylogenetic analysis

**Figure 2:**
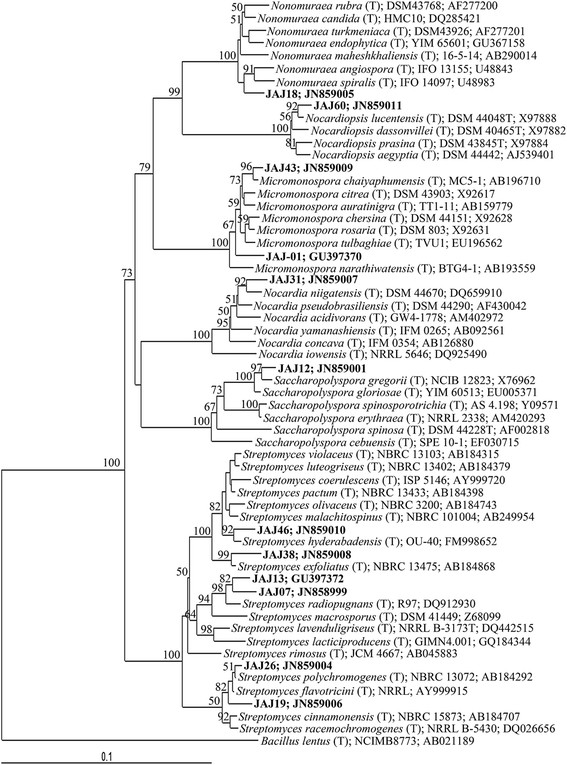
**Neighbour-joining phylogenetic tree inferred from 16S rRNA gene sequences.** The phylogenetic tree shows the phylogenetic relationship of isolates with related genera. Bootstrap values are expressed as percentages of 1000 replications. Bootstrap values, >50% are shown at branch points. *Bacillus lentus*^T^ (GenBank accession number AB021189) was used as out-group. Score bar represents 1 nucleotide substitution per 100 nucleotides.

16S rDNA of 12 representative isolates belonging to different clusters established by ARDRA was sequenced and used to determine the diversity among the isolates. These representatives were affiliated with one of the following five genera belonging to Actinobacteria (Figure 2).

#### Streptomyces

The most abundant group of isolates was affiliated with the genus *Streptomyces*, represented by six ribotypes accounting for 48% of the total actinomycete population. Of these ribotypes JAJ07 and JAJ13 fell in a same clade in the phylogenetic tree with 99% and 98% sequence similarities respectively to nearest type strain *Streptomyces radiopugnans* (GenBank: DQ912930), representing 18% of total isolates. The other ribotypes of *Streptomyces,* JAJ19 (GenBank: JN859006) (representing 9% of isolates) and JAJ26 (GenBank: JN859004) (representing 4% of all isolates) were closely related to type strain *Streptomyces flavotricini* (GenBank: AY999915) with 99.9% sequence similarity and *Streptomyces polychromogenes* (GenBank: AB184292) with 99.9% sequence similarity, respectively. The ribotype JAJ46 (GenBank: JN859010) was clustered with *Streptomyces hyderabadensis* (GenBank: FM998652) with 98% of sequence identity, representing 4% of all actinomycete isolates. Likewise, the ribotype JAJ38 (GenBank: JN859008) was clustered with *Streptomyces exfoliatus* (GenBank: AB184868) by 99.5% sequence identity, representing 13% of all isolates.

#### Micromonospora

The second most dominant group in the isolates was the genera *Micromonospora,* represented by two different ribotypes accounting for 23% of total actinomycete isolates. Ribotype JAJ43 (GenBank: JN859009) was 99% identical to type strain *Micromonospora chaiyaphumensis* (GenBank: AB196710), representing 13% of the entire actinomycete population. Ribotype JAJ01 (GenBank: GU397370) was related to type strain *Micromonospora tulbaghiae* (GenBank: EU196562) with 99% sequence similarity, representing 10% of all actinomycetes isolates.

#### Nocardia

This genus was represented by ribotype JAJ31 (GenBank: JN859007), representing 15% of all actinomycete isolates, showed 98% sequence similarity to type strain *Nocardia niigatensis* (GenBank: DQ659910).

#### Nonomuraea

The ribotype JAJ18 (GenBank: JN859005) was clustered with type strains *Nonomuraea spiralis* (GenBank: U48983, 98.5% 16S rDNA sequence identity) and *Nonomuraea angiospora* GenBank: (U48843, 98% 16S rDNA sequence identity)*,* representing 7% of all actinomycete isolates.

#### Nocardiopsis

The genus *Nocardiopsis* was also represented by only one phylotype. The ribotype JAJ60 (GenBank: JN859011), representing 4% of all actinomycete isolates, shared 99% sequence similarity with type strain *Nocardiopsis lucentensis* (GenBank: X97888).

#### Saccharopolyspora

The ribotype JAJ12 (GenBank: JN859001) was identified as *Saccharopolyspora* species, representing 3% of all actinomycete isolates, showed 99% sequence similarity to type strain *Saccharopolyspora gregorii* (GenBank: X76962).

### Morphology of actinomycete isolates

**Figure 3:**
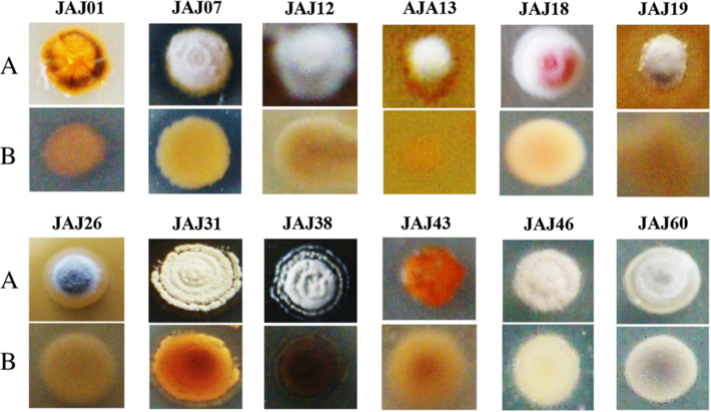
**Colony morphology of representative actinomycete isolates derived from solar saltern ponds.** ‘**A**’ represents the colony view from aerial mycelium side and ‘**B**’ is the substrate mycelium view. Colonies were distinguished based on the colour of aerial and substrate mycelia and sporulation.

Morphology of representative isolates was visually observed and their aerial and substrate mycelium are shown in Figure 3. The isolates JAJ13, JAJ07, JAJ19, JAJ26, JAJ38 and JAJ46 grew well on IM2 with characteristic musty odour, spore formation, dimorphic mycelial forms as aerial and subterranean mycelium and non-motile colonies. All these streptomycete isolates attained maximum growth with grey to white coloured sporulation after three days of incubation. Isolates JAJ01 and JAJ43, lacking aerial mycelium on IM2, grew with pigmentation that ranged from bright to pale orange to black and pale to golden yellow colour respectively.

Isolate JAJ18 which was identified as *Nonomuraea species,* produced disc shaped pink colour colonies with sporulation largely at the edges of the colony. Isolate JAJ31, identified as *Nocardia* species, produced cream color aerial mycelium and wheat colour substrate mycelium. *Saccharopolyspora* strain, JAJ12 produced snow white aerial mycelium and wheat colour substrate mycelium. Isolate JAJ60, identified as *Nocardiopsis* species produced grey aerial mycelium and substrate mycelium with moderate sporulation on IM2.

## Discussion

Hypersaline multi-pond solar salterns are renowned as a habitat for halophiles [15-17,19,20] and an understanding of the microbial assemblages in such hypersaline environments is highly desirable due to their potential applications [5,6,11]. In this study, selective isolation techniques employing heat treatment and stamping have been used to assess actinomycetes population in soil sediments from the solar salterns. The role of selective isolation procedures is often vital for isolation of rare and novel actinomycetes [23,24]. A combination of stamping method and low nutrient agar medium was described as efficient method for the isolation of novel actinomycetes from marine sediments [23]. Among the 69 isolates, most of the isolates including *Nonomuraea* and *Micromonospora* were obtained from low nutrient IM4 plates stamped with dried samples, even though sediment-processing methods were not applied equally to all samples. The low nutrient medium slowed down the growth of fast growing Gram negative bacteria and the stamping favoured the germination of actinomycete spores adhered to soil particles. The antibiotics used with agar medium also favoured the growth of actinomycetes by preventing the growth of fungal strains and other contaminants. In a nutshell, the selective isolation methods favoured the isolation of variety of actinomycetes inclusive of *Nonomuraea* species from the solar saltern.

The microbial phylogenetic diversity among the isolated actinomycetes was studied based on their 16S rDNA. ARDRA was employed in this study to assist in distinguishing among the taxonomic groups. The ARDRA is an extension of RFLP technique, commits the four cutter restriction enzymes to discriminate the microbes at inter genus and intra genus levels [25-28]. The restriction enzyme, *HaeIII* recognizes specific tetranucleotide sites available frequently in DNA, widely used for discrimination of bacterial species [29,30]. In the present study, twelve actinomycete isolates were selected as representative strains from the entire actinomycete population using ARDRA with *HaeIII*. The 16S rDNA sequence data of isolates other than the selected representatives revealed that some different species of same genus showed same ARDRA pattern but species of different genus did not show the same ARDRA pattern and it conformed the coverage of all genera encompassed by the entire actinomycetes population, even though all the species were not covered. In other words the ARDRA performed with *HaeIII* successively have discriminated the isolates at genus level and only to some extent the species level.

Phylogenetic analysis based on 16S rRNA gene sequences of representative isolates revealed the presence of actinomycetes affiliated to five genera; *Streptomyces, Micromonospora, Nocardia, Nonomuraea, Saccharopolyspora,* and *Nocardiopsis* in saltern soil samples. The most abundant actinomycete group detected was allocated to the genus *Streptomyces* followed by *Micromonospora*. The predominance of the *Streptomyces* followed by *Micromonospora* has already been observed previously in saline soils [31]. The *Nocardia* accomplish the third position of predominance among the all actinomycete isolates. Occurrence of *Nocardia* in hypersaline environment has previously been reported by Quesada *et al*. [32].

Existence of *Nonomuraea, Saccharopolyspora* and *Nocardiopsis* were also found in the saltern ponds. Previously the genus *Nonomuraea* has been reported from various soil samples including arid soil, cave soil and mangrove rhizosphere [33-35]. However, there are no reports on isolation of *Nonomuraea* species from saline lakes or solar saltern ponds and also from India. The other two genera, *Saccharopolyspora* and *Nocardiopsis* have frequently been isolated from salt lakes and saline soils [36,37].

## Conclusion

In this study, actinomycete diversity was explored from the Indian coastal solar salterns and the isolates recovered were assigned to five genera on the basis of their 16S rDNA sequences and this is the first step towards better understanding of actinomycete community from solar saltern ponds in Tuticorin, India. In a nutshell, the results obtained reveal the significant actinomycete diversity and further researches including the bioactive assays and molecular characterizations are needed to clarify the biotechnological importance and ecological roles of this actinomycete population.

## Methods

### Sample collection and physico-chemical analysis

**Figure 4:**
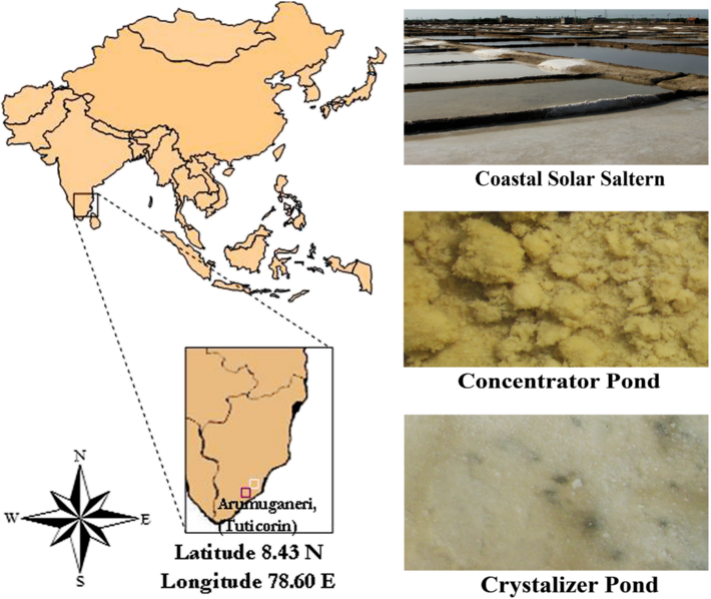
**Map of the location of the sampling site and appearance of coastal solar saltern ponds.** Tuticorin is one of the major salt production sites in India. Soil samples were collected from both the concentrator pond and crystallizer pond of the solar saltern for this work.

Saltpan sediment samples were collected aseptically from two different salinity ponds: concentrator ponds and crystallizer ponds of solar salterns from Tuticorin (about Latitude 8.43 N and Longitude 78.60E) in India (Figure 4). The collected samples were subjected to physiochemical analysis following standard methods. Soil pH and electrical conductivity (EC) were measured using a soil/water suspension (1:2 w/v) with potentiometer and conductimeter respectively according to Jackson [38]. The saltpan mud soil sample was air dried and organic matter [organic carbon] was determined according to the method of Walkley and Black [39]. Total nitrogen was determined by the micro kjeldahl method [38]. Total Phosphorous was estimated according to Pemberton [40]. Total sodium and potassium was estimated using atomic absorption spectrophotometer (AA-6200, Shimadzu, Japan). The total nitrogen, phosphorus and potassium estimations were carried out after the acid digestion of soil samples. Micronutrients, Zn, Cu, Fe and Mn were determined using flame photometry. 

### Sample processing and selective isolation

Ten composite samples were made from five samples collected randomly from each pond of the solar saltern. The composite samples were homogenized and portions processed by one or another of the following two selective methods modified from the earlier methods described by Gontang *et al*. [23] to reduce the numbers of Gram-negative bacteria and to enrich slow-growing, spore-forming actinomycetes. In the first processing method, 10 g of wet sediment sample was dried overnight in a laminar flow hood, ground lightly with a sterile pestle and stamped onto the agar media using sterile cotton plug, creating a serial dilution effect. In the second processing method (dilution-and-heat-shock method), 1 gm of the sample was suspended in 4 ml of sterile water, heated for 6 min at 55°C, vigorously shaken and further diluted (1:4) in sterile water, and 50 μl of each dilution was spread with a sterile glass rod onto agar-based isolation media.

Isolation media contained the following components: IM1[41], 10 g of starch, 4 g of yeast extract, 2 g of peptone, 18 g of agar, and 1 liter of natural seawater; IM2 [11], 10 g of starch, 4 g of yeast extract, 5 g of NaCl, 2 g of NH_4_SO_4_, 1 g of MgSO_4_, 1 g of K_2_HPO_4_, 22 g of agar, and 1 liter of distilled water; IM3 (Starch Casein Salt Agar) [42], 10 g of Starch, 0.3 g of casein, 2 g of KNO_3_, 4.6 g of sodium chloride, 2 g of K_2_HPO_4_, 0.05 g of MgSO_4_, 0.02 g of CaCO_3_, 0.01 g of FeSO_4_, 1 mg of Zn(O_2_CCH_3_)_2,_ 18 g of agar and 1 litre of distilled water; and IM4 [41], 6 g of glucose, 2 g of chitin, 18 g of agar, and 1 liter of natural seawater. All isolation media were amended with filter-sterilized cycloheximide (100 μg/ml) and nalidixic acid (5 μg/ml) to favor the selective isolation of actinomycetes. Inoculated plates were incubated at 30°C for up to 6 weeks, and all leathery colonies were removed from the original isolation plates and sub-cultured on IM2.

### 16S rDNA amplification

Isolates were grown in trypticase soy broth supplemented with 1-2% NaCl [w/v] or NDYE medium according to their growth in 150 ml conical flasks for 3–5 d. The mycelia were harvested from the broth by centrifugation at 10,000 rpm for 3 min and washed twice with sterile milli Q water. From the harvested mycelia, the DNA was extracted following standard phenol-chloroform extraction procedure [43].

The 16S rDNA was amplified from genomic DNA samples using eubacterial universal primers 27 F 5^′^-AGAGTT TGA TCC TGG CTC AG-3^′^ and 1492R 5^′^-GGT TAC CTT GTT ACG ACT T-3^′^[44]. The amplification was carried out in a 50 μL volume by using 25 ng of genomic DNA as template with 1X reaction Buffer (10 mM Tris pH 8.3, 50 mM KCl, 1.5 mM MgCl_2_), 200 μM of each dNTP, 10 pM of each primer and 0.05 U of Taq DNA polymerase (Sigma, USA). The thermal cycling conditions were as follows: initial denaturation at 94°C for 5 min; 31 cycles at 95°C for 30 s, 54°C for 90 s and 72°C for 120 s; and a final extension at 72°C for 5 min. The amplification reaction was performed by Bio-Rad thermal cycler (MyCycler, Bio-Rad, USA) and the amplified products were examined by 1% agarose gel electrophoresis.

### Amplified ribosomal DNA restriction analysis (ARDRA)

To identify the number of polymorphic groups and select representative strains among the actinomycete isolates, aliquots of purified 16S rDNA amplicons were subjected to amplified ribosomal DNA restriction analysis. The purified PCR products were digested separately with *Hae*III and *Hinf* I, in 20 μL reaction volume by using manufacture’s recommended buffer and temperature. The digested restriction fragments were electrophoresed in 2.5% agarose gels using TE buffer. The gels were stained with ethidium bromide and visualized under UV transilluminator. Strong and clear bands were scored in a binary data form and used for similarity and clustering analysis in numerical taxonomy analysis program package, NTSYS-pc 2.02i [45]. Similarities among the isolates were calculated by Jaccard’s coefficient [46] and the dendrogram was constructed using UPGMA method [47].

### 16S rDNA sequencing and phylogenetic analysis

The PCR amplicons of 12 actinomycete isolates with representative ARDRA profiles were purified using PCR product purification spin kit (HiPurA, HiMedia, India) and sequenced by Applied Biosystems 3730XL DNA Analyzer using same primer set as used in PCR amplification. In addition to chosen representative isolates, 16S rDNA of 10 other actinomycete isolates that showed ARDRA patterns identical to representative isolates were also sequenced to validate the ARDRA. All the sequences obtained from sequencing were analyzed and edited by using BioEdit software [48]. Initially all the 16S rRNA sequences were compared to sequences in GenBank by use of the Basic Local Alignment Search Tool online service to determine approximate phylogenetic position [49]. Sequences then were aligned using ClustalX [50] with representative actinomycete16S rRNA gene sequences. Phylogenetic tree was inferred by using suitable programs of the PHYLIP (phylogeny inference package) version 3.68 [51].

### Morphology of representative strains

All the representative isolates obtained from ARDRA analysis were subjected to morphological characterization on IM2. Inoculated plates were incubated at 29°C for 7 to 10 days and their morphology was visually observed.

### Nucleotide sequence accession numbers

The 16S rRNA gene sequences of representative strains have been deposited in the GenBank database under the accession numbers GenBank: JN858999 - GenBank: N859013 and GenBank: GU397370 - GenBank: GU397372.

## Competing interests

Both authors declare that they have no competing interests.

## Authors’ contributions

PAJ and Prof. Solomon collected samples from Tuticorin, India on 23^rd^ March 2010. PAJ carried out all the experiments, analyzed data, and drafted the manuscript under the complete guidance of Prof. Solomon. Both the authors read and approved the final manuscript.
